# Comparing a knowledge-driven approach to a supervised machine learning approach in large-scale extraction of drug-side effect relationships from free-text biomedical literature

**DOI:** 10.1186/1471-2105-16-S5-S6

**Published:** 2015-03-18

**Authors:** Rong Xu, QuanQiu Wang

**Affiliations:** 1 Case Western Reserve University, Cleveland OH 44106, USA; 2 ThinTek, LLC, Palo Alto CA 94306, USA

## Abstract

**Background:**

Systems approaches to studying drug-side-effect (drug-SE) associations are emerging as an active research area for both drug target discovery and drug repositioning. However, a comprehensive drug-SE association knowledge base does not exist. In this study, we present a novel knowledge-driven (KD) approach to effectively extract a large number of drug-SE pairs from published biomedical literature.

**Data and methods:**

For the text corpus, we used 21,354,075 MEDLINE records (119,085,682 sentences). First, we used known drug-SE associations derived from FDA drug labels as prior knowledge to automatically find SE-related sentences and abstracts. We then extracted a total of 49,575 drug-SE pairs from MEDLINE sentences and 180,454 pairs from abstracts.

**Results:**

On average, the KD approach has achieved a precision of 0.335, a recall of 0.509, and an F1 of 0.392, which is significantly better than a SVM-based machine learning approach (precision: 0.135, recall: 0.900, F1: 0.233) with a 73.0% increase in F1 score. Through integrative analysis, we demonstrate that the higher-level phenotypic drug-SE relationships reflects lower-level genetic, genomic, and chemical drug mechanisms. In addition, we show that the extracted drug-SE pairs can be directly used in drug repositioning.

**Conclusion:**

In summary, we automatically constructed a large-scale higher-level drug phenotype relationship knowledge, which can have great potential in computational drug discovery.

## Introduction

It has been increasingly recognized that similar side effects of seemingly unrelated drugs can be caused by their common off-targets and that drugs with similar side effects are likely to share molecular targets [1]. Therefore, systems approaches to studying side effect relationships among drugs and integration of this drug phenotypic data with drug-related genetic, genomic, proteomic, and chemical data will facilitate drug target discovery and drug repositioning. The availability of a comprehensive drug-side effect (SE) relationship knowledge base is critical for these tasks. Current drug phenotype-driven systems approaches rely exclusively on drug-SE associations extracted from FDA drug labels. However, there exists a large amount of additional drug-SE relationship knowledge in the large body of published biomedical literature. In this study, we present a novel knowledge-driven approach to automatically extract a large number of drug-SE pairs from 21 million published biomedical abstracts. We systematically analyzed extracted drug-SE pairs in combination with drug-related gene targets, metabolism, pathways, gene expression and chemical structure data. We show that these extracted drug-SE pairs have great potential in drug discovery.

## Background

Systems approaches to studying the phenotypic relationships among drugs can facilitate rapid drug target discovery and drug repositioning. Computational approaches to predicting drug targets have often been based on chemical similarity measures and docking strategies [7,16]. Similarly, many computational strategies for drug repositioning have been explored [6]. The majority of these approaches leverage on known drug properties such as chemical similarity [7], molecular activity similarity [12], molecular docking [8], and gene expression profile similarity [13]. In a seminal paper, Campillos et al. used phenotypic side-effect similarities among drugs to predict new targets for drugs [1]. However, their analysis was limited to drug-SE relationships derived solely from the FDA drug labels. In one of our recent studies, we show that much of the drug-SE association knowledge from biomedical literature has not been captured in FDA drug labels yet [17].

Currently, more than 21 million biomedical records are available on MEDLINE. While many biomedical relationship extraction tasks have focused on extracting relationships between drugs, diseases, proteins, or genes [2,18,19], extracting drug-SE relationships from MEDLINE has been less explored. Recently, Gurulingappa et al. trained and tested a supervised machine learning classifier to classify drug-condition pairs in a set of 2972 manually annotated case reports [4]. That study focused on a limited set of drugs and side effects and case reports. It is unclear how their approach can be effectively scaled up to the whole MEDLINE in building a large-scale drug-SE relationship knowledge base. Recently, we developed an approach in boosting drug safety signal detection from FDA Adverse Event Reporting System (FAERS) using evidence from MEDLINE [20]. We developed an automatic approach to extract anticancer drug-specific side effects from MEDLINE by developing specific filtering and ranking schemes [21]. We developed a pattern-based learning approach to accurately extract drug-SE pairs from MEDLINE sentences [22]. We combined automatic table classification and relationship extraction in extracting anticancer drug-side effect pairs from full-text articles [23]. In this study, we present a knowledge-driven (KD) text-classification-based approach to extract drug-SE pairs from MEDLINE sentences. Different from our previous studies where we extracted drug-SE pairs from unclassified sentence, here we classified sentences into drug-SE-related and -unrelated before relationship extraction. Our approach is also different from other text classification-based approaches that often trained text classifiers using annotated training datasets to find drug-SE-related sentences [4], instead, we implicitly classified MEDLINE sentences using known drug-SE pairs. Since our study did not explicitly train a text classifier, it is highly dynamic and effective: it can easily incorporate any changing prior knowledge and quickly extracted drug-SE pairs from the whole MEDLINE (21,354,075 abstracts and 119,085,682 sentences).

## Approach

Our study is based on the two key observations: (1) multiple side effects for a drug are often reported in the same sentences or abstracts; and (2) if a sentence contains a known drug-SE pair, then this sentence is likely to be SE-relevant. Other pairs in this SE-related sentence are likely to be drug-SE pairs. For example, the sentence "*At the final **irinotecan **dose of 50 mg/m(2), grade 3 or higher toxicity included **diarrhea **(26%), **neutropenia **(21%), **nausea **(18%), **fatigue **(16%), **anorexia **(13%), and **thrombosis/embolism **(13%)*" (PMID 19139178) contains a known drug-SE pair "irinotecan-diarrhea." Based on this fact, we know that this sentence is SE-related and that the other five pairs in this sentence are likely to be drug-SE pairs. On the other hand, the following sentence "*Weekly docetaxel, cisplatin, and irinotecan (TPC): results of a multicenter phase II trial in patients with metastatic esophagogastric cancer *" contain no known drug-SE pair, therefore no pair will be extracted from this sentences even though it contains three drug-disease pairs. In this study, we used all known drug-SE pairs derived from FDA drug labels as prior knowledge to find SE-related MEDLINE sentences and abstracts, from which many additional drug-SE pairs that have not included in FDA drug labels are then extracted. We compared the KD approach to a support vector machine (SVM)-based approach.

## Data and methods

The entire experimental process consists of the following steps: (1) build a local MEDLINE search engine; (2) develop, evaluate and compare the KD approach to a SVM-based approach; (3) extract drug-SE pairs from MEDLINE; and (4) systematically analyze the correlation between drug-associated side effects and drug gene targets, metabolism genes, chemical similarity, and disease indications.

### Build a local MEDLINE search engine

We downloaded a total of 21,354,075 MEDLINE citations (119,085,682 sentences) published between 1965 and 2012 from the U.S. National Library of Medicine http://mbr.nlm.nih.gov/Download/index.shtml. Each sentence was syntactically parsed with Stanford Parser [9] using the Amazon Cloud computing service (a total of 3,500 instance-hours with High-CPU Extra Large Instance used). We used the publicly available information retrieval library Lucene http://lucene.apache.org to create a local MEDLINE search engine with indices created on both sentences and their corresponding parse trees. We have recently used this local search engine in our recent tasks of extracting disease-manifestation relationships [19] and anticancer drug-SE pairs from MEDLINE [21].

### Develop, evaluate and compare the KD approach to the SVM-based approach

We downloaded a total of 100,049 known drug-SE pairs from SIDER (Side Effect Resource), a public, machine-readable side effect resource that was automatically constructed from FDA package inserts [10]. These pairs are used as prior knowledge for the KD approach. Using each drug-SE pair from the prior knowledge data as a search query to the local MEDLINE search engine, we retrieved all MEDLINE sentences and abstracts that contain at least one known drug-SE pair. These sentences are determined as SE-related. We then extracted drug-SE co-occurrence pairs from these SE-relevant sentences, with the restriction that both drug and SE names must be noun phrases in the parse trees of the sentences. We have recently shown that this restriction can increase the precision of biomedical relationship extraction from MEDLINE [19-22]. The drug and SE lists (996 drugs (generic names) and 4,199 SE terms) are from SIDER.

**Figure 1:**
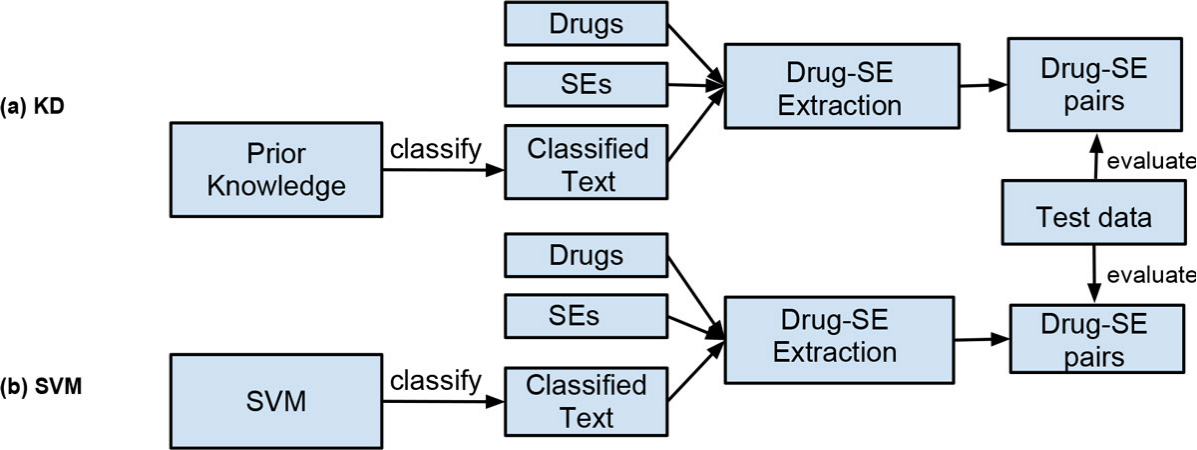
**KD approach vs. SVM approach**.

We compared the drug-SE extraction from sentences classified using the KD approach with that from sentences classified using a SVM-based text classifier (described later). For comparison, we selected ten drugs from SIDER that are associated with the most numbers of SEs and compared the performance of KD approach to the SVM approach in extracting drug-SE pairs for each of them (Figure 1). For each drug, we randomly split its drug-SE pairs into two equal parts: training dataset and testing dataset. For the KD approach, we first retrieved all sentences that contain at least one of the 10 drugs and at least one SE term from the SE lexicon (4,199 SE terms). We then classified these sentences into SE-related and -unrelated. A sentence is determined as SE-related if it contains at least one known drug-SE pair from the training dataset (prior knowledge). We then extracted additional drug-SE pairs from these sentences.

For the SVM-based approach, we first classified MEDLINE sentences into drug-SE-related and -unrelated using a pre-trained SVM-sentence classifier. We then extracted drug-SE co-occurrence pairs from positively classified sentences. A two-class SVM-based sentence classifier was trained using implementation in WeKa [5]. The positive training data consisted of a total of 320,175 sentences, each of which contained at least one known drug-SE pairs from SIDER (the testing data for above 10 drugs were excluded). Equal number of negative sentences was randomly selected from the rest of MEDLINE sentences. The SVM-based sentence classifier used polynomial kernel, bag-of-words feature, TF-IDF weighting, stemming and stopwords-removal. The bag-of-words feature was used since it is often the case that the appearance of one specific word such as 'toxicity' can be used to determine whether a sentence is drug-SE-related. The 10-fold cross validation was used in training the SVM classifier. For both KD and SVM-based approaches, the input sentences are the same, which are sentences that contain at least one of the 10 drugs and at least one term from the SE lexicon. We evaluated and compared the performance using the same testing datasets. Precisions, recalls and F1 scores for these 10 drugs were calculated.

### Extract drug-SE pairs from 21 Million MEDLINE records

After evaluating the KD-approach using 10 drugs, we then scaled this approach to extract drug-SE pairs from all MEDLINE sentences and abstracts. We used all 100,049 drug-SE pairs from SIDER as prior knowledge to classify all MEDLINE sentences and abstracts into SE-related and -unrelated. For the classification, we used each of the 100,049 drug-SE pairs from SIDER as a search query to the local MEDLINE search engine. Both sentences and abstracts containing the pair were retrieved as SE-related. Sentences that are not retrieved are assumed to be SE-unrelated and ignored. In total, we extracted 49,575 drug-SE pairs from sentences and 180,454 pairs from abstracts using the drug and SE lists (996 drugs and 4,199 SE terms) we compiled from SIDER. These extracted drug-SE pairs were used in the subsequent semantic analysis.

### Analyze the correlations between drug-associated side effects and genetic, genomic and chemical drug properties

Many current computational approaches for drug target discovery [7,16] and drug repositioning[7,12,8,13] used only lower-level genetic, genomic, and chemical drug properties. In this study, we investigated whether the large number of higher-level phenotypic drug-SE relationship data that we extracted from MEDLINE implicitly captured lower-level drug mechanism, therefore can be leveraged for drug target discovery and drug repositioning. In extracting drug-SE pairs from MEDLINE, we used the generic names of FDA-approved drugs. For the correlation analysis, we use drug generic names to link drug-SE pairs to drug-related information from different databases.

#### Correlation with drug target genes

Drug side effects are often caused by drugs acting on their target genes. We investigated whether drug-drug pairs that shared SEs tend to share gene targets. We downloaded a total of 13,635 drug-target gene associations from DrugBank [15], a knowledge base for drugs, drug actions and drug targets. The drug-gene pairs are comprised of 3,454 drugs and 1,816 genes. We first mapped drugs of drug-SE pairs extracted from MEDLINE to drugs of drug-gene pairs from DrugBank. For drug-drug pairs that share SEs at different cutoffs, we calculated the average number of shared gene targets.

#### Correlation with drug metabolizing genes

Drug metabolism plays critical role in drug-associated side effects. We investigated whether drug-drug pairs that shared SEs also share drug metabolism genes. We downloaded a total of 4,399 drug-gene pairs from PharmGKB [14], a repository of drug pharmacogenetics information. For drug-drug pairs that share SEs at different cutoffs, we calculated the average number of shared metabolism genes.

#### Correlation with genetic, genomic and chemical drug-drug relationships

Drug related pathway, genomic and chemical relationship information was obtained from STITCH [11], a resource of known and predicted interactions of chemicals and proteins. In STITCH, chemicals are linked to other chemicals and proteins by four types of relationships: chemical reactions from manually curated pathway databases ("Database"), literature associations ("Textmining"), similar 2D structures ("Similarity") and similar activities ("Experimental") based on drug-induced perturbation on the gene expression level [12]. We used chemical-chemical relationships from curated pathway database ("Database", 342,072 chemical-chemical pairs), chemical 2D structure ("Similarity", 607,588 chemical-chemical pairs) and gene expression ("Experimental", 238,380 chemical-chemical pairs). The text mining-based co-occurrence pairs were not used since they provide no explicit semantic relationships for chemical-chemical pairs. For drug-drug pairs that share SEs at different cutoffs, we calculated the average chemical similarity scores.

#### Correlation with disease indications

If high-level phenotypic drug-SE relationships implicitly capture known and unknown drug-related genetic, genomic and chemical information, then drug-SE pairs may be directly used for drug repositioning, as suggested in a recent review article [6]. In this study, we investigated whether drug-drug pairs that shared SEs tend to share disease indications. We recently extracted a total of 52,000 drug-disease pairs from ClinicalTrials.gov [24], a registry of federally and privately supported clinical trials conducted in the United States and around the world. The drug-disease pairs contain 2,035 drugs and 9,591 diseases. For drug-drug pairs that share SEs at different cutoffs, we calculated the average number of shared disease indications.

## Results

### The KD approach is more effective than the SVM-based approach in extracting drug-SE pairs from MEDLINE

As shown in Table 1 the KD-based approach performed consistently better than the SVM-based approach for all 10 drugs. On average, KD achieved a precision of 0.335, a recall of 0.509 and an F1 of 0.395. The SVM-based approach had an average precision of 0.135, a recall of 0.900 and an F1 of 0.233. The F1 of the KD approach is 73.0% higher than that of the SVM-based approach. However the reported precision and recall may significantly underestimate the actual precision and recall for drug-SE pairs that we extracted from MEDLINE. For these 10 drugs, we used half of their drug-SE pairs from SIDER as testing data, however, drug-SE association knowledge in SIDER (derived from FDA drug labels) and from MEDLINE has been shown to be largely complementary [17]. In order to accurately evaluate drug-SE extractions from MEDLINE. It will be necessary to construct a MEDLINE-based gold standard.

**Table 1:** Compare knowledge-driven approach (KD) to SVM for ten drugs

Drug	KD			SVM			F1 Increase
		
	Precision	Recall	F1	Precision	Recall	F1	
Fluoxetine	0.370	0.425	0.395	0.178	0.838	0.294	34.4%
Tacrolimus	0.315	0.519	0.392	0.140	0.961	0.245	60.0%
Risperidone	0.561	0.622	0.590	0.234	0.959	0.377	56.5%
Carbamazepine	0.341	0.627	0.442	0.118	0.896	0.209	111.5%
Ibuprofen	0.230	0.406	0.294	0.106	0.891	0.189	55.6%
Olanzapine	0.463	0.492	0.477	0.180	0.921	0.301	58.5%
Morphine	0.214	0.611	0.317	0.090	0.833	0.163	94.5%
Phenytoin	0.229	0.403	0.292	0.104	0.881	0.186	57.0%
Methotrexate	0.230	0.607	0.333	0.082	0.984	0.152	119.1%
Ciprofloxacin	0.397	0.377	0.387	0.121	0.836	0.212	82.5%
**Average**	**0.335**	**0.509**	**0.392**	**0.135**	**0.900**	**0.233**	**73.0%**

### Drug-associated side effects positively correlate with drug-associated gene targets

**Figure 2:**
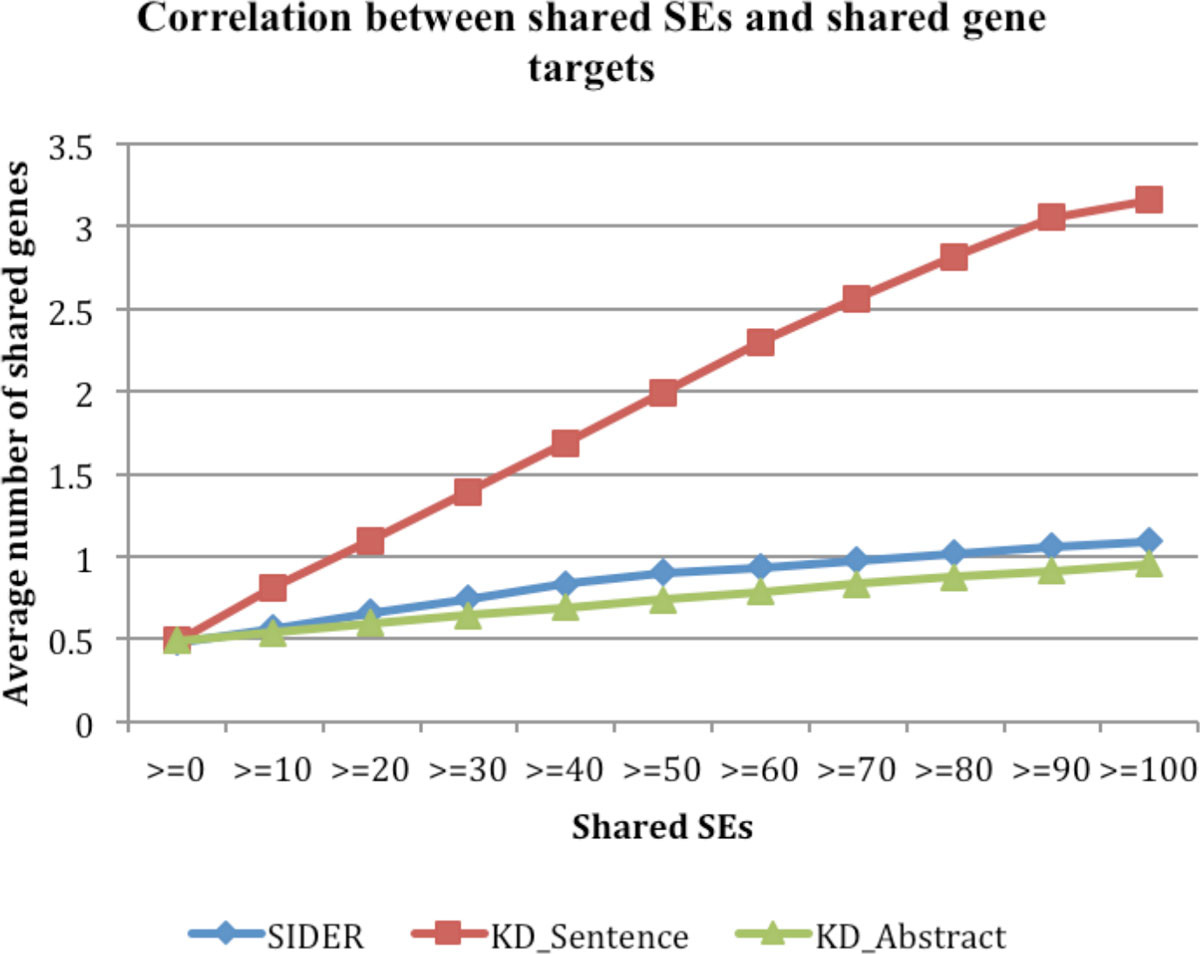
**The correlations between SEs and gene targets for drug-SE pairs from: SIDER, MEDLINE sentences ("KD_Sentence"), and abstracts("KD_Abstract")**.

We investigated whether drug-drug pairs that share SEs tended to share gene targets. As shown in Figure 2, there is positive correlation between SEs and gene targets, with the positive correlation being much stronger for drug-SE pairs extracted from MEDLINE sentences than those from SIDER or MEDLINE abstracts. For instance, the average number of shared gene targets for all drug-drug pairs is 0.492. The number significantly increased to 0.813 for drug-drug pairs that shared at least one SEs and to 3.161 for pairs that shared at least 100 SEs. This strong positive correlation indicates that we may use these extracted drug-SE pairs to discover novel drug targets or use drug-related gene targets to predict unknown drug side effects.

### Drug-associated side effects positively correlate with drug-associated metabolism genes

**Figure 3:**
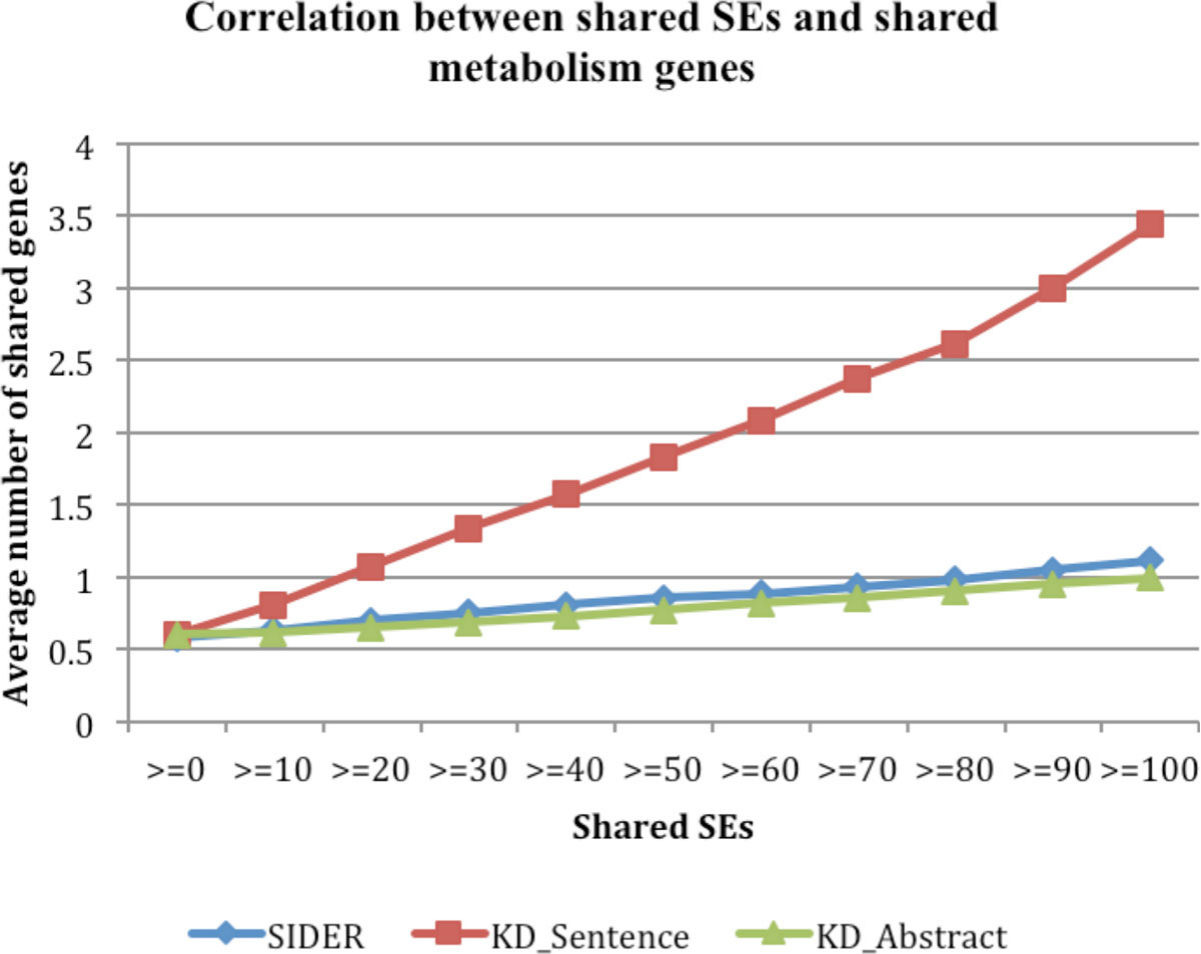
**The correlations between SEs and drug metabolism genes for drug-SE pairs from: SIDER, MEDLINE sentences ("KD_Sentence"), and abstracts("KD_Abstract")**.

Drug-associated side effects are closely related to drug metabolism. As shown in Figure 3, drug-drug pairs that share SEs tended to share more metabolism genes. For instance, the average number of shared drug metabolism genes for all drug-drug pairs is 0.605. The number significantly increased to 0.809 for drug-drug pairs that shared at least one SEs and to 3.441 for drug-drug pairs sharing at least 100 SEs. This strong positive correlation indicates that we may achieve personalized medicine (in terms drug toxicity) by predicting patient-specific drug side effects based on their genotype profiles.

### Drug-associated side effects positively correlate with drug-associated pathways and gene expression

**Figure 4:**
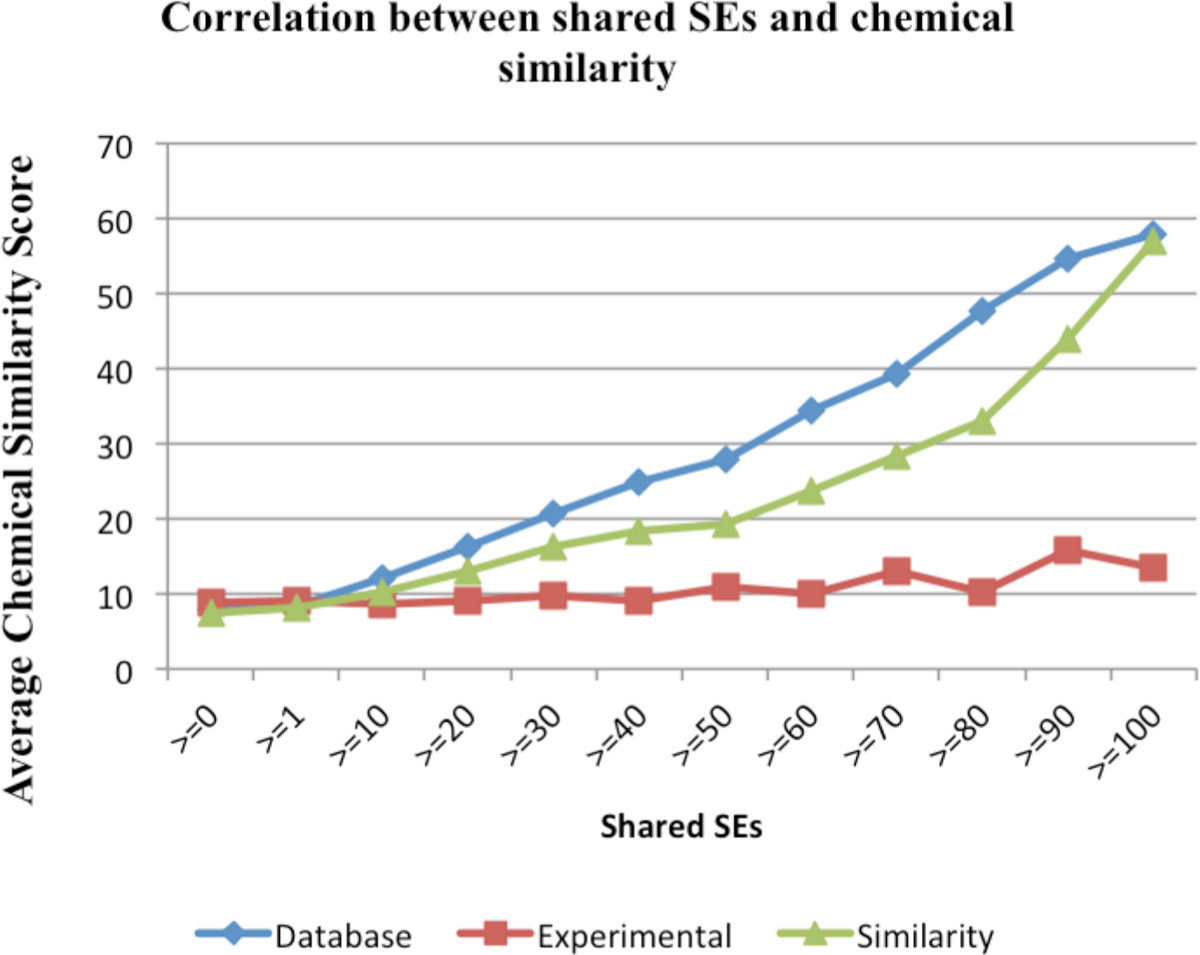
**The correlation between shared SEs and chemical similarity: "Database", "Experimental" and "Similarity"**.

Genetic, genomic and structural chemical-chemical relationships have been widely used for both drug target discovery and drug repositioning. We investigated whether the phenotypic side effect similarity between drugs captured chemical similarities as measured by chemical structure, gene co-expression and pathway interactions. We used chemical-chemical relationships from curated pathway database ("Database"), chemical 2D structure ("Similarity") and gene expression ("Experimental") in our study. As shown in Figure 4, drug-drug pairs that share SEs tend to share common pathway and 2D chemical structure, but not gene co-expression profiles. For instance, the average number of chemical similarity score based on "Database" for all drug-drug combination is 7.898; the number significantly increased to 12.06 for drug-drug pairs that share at least 10 SEs and to 27.795 for pairs that share at least 50 SEs. The correlation curve for chemical structure-based chemical relationships is similar but less prominent. There is no obvious correlation between gene expression-based drug similarity and drug side effects. In summary, high-level phenotypic relationships among drugs as determined by shared side effects indeed reflect drug relationships at genetic and chemical levels. Hence, systematic approaches in studying these higher-level phenotypic drug relationships can reveal insights into drug molecular mechanisms and offer opportunities for drug target discovery and drug repositioning.

### Drug-associated side effects positively correlate with drug-associated disease indications

**Figure 5:**
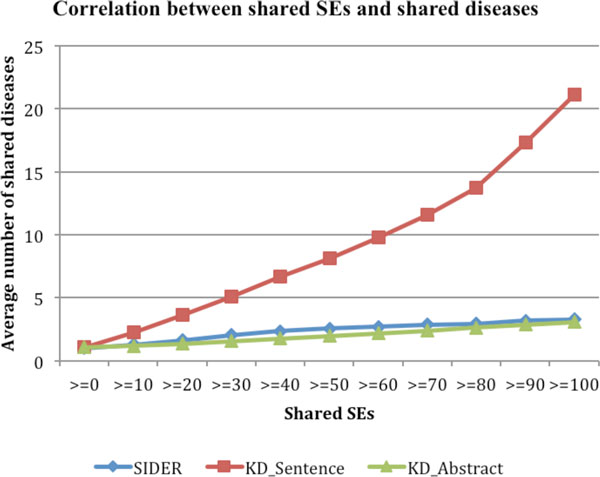
**The correlation between SEs and disease indications for drug-SE pairs from: SIDER, MEDLINE sentences ("KD_Sentence"), and abstracts ("KD_Abstract")**.

In previous sections, we have shown that drug-associated side effects reflect drug-associated molecular mechanism. In this section, we investigated whether the large number of drug-SE pairs that we extracted from MEDLINE have potential in being directly used in drug repositioning. As shown in Figure 5, drug-drug pairs that share SEs share significantly more disease indications than pairs sharing no SEs. The positive correlation is much stronger when drug-SE pairs extracted from MEDLINE sentences were used than pairs from SIDER or MEDLINE abstracts. For instance, the average number of shared drug indications for all possible drug-drug pairs is 1.074. The number significantly increased to 2.281 for pairs that shared at least one SEs and to 21.164 for drug-drug pairs sharing at least 100 SEs. This strong positive correlation between drug-associated SEs and drug-associated disease treatments indicates that we may directly use the observed drug side effects for drug repositioning. For example, if two drugs often have the same side effects when used in patients, we can reason that these two drugs have similar underlying mechanisms (share genetic targets, pathways, gene expression or chemical structures etc) and can be used to treat the same diseases. The advantage in using high-level phenotypic drug side effect information compared to lower-level genetic and chemical information is that is that we can reposition existing drugs for new disease indications without knowing underlying drug mechanisms or disease etiology, which largely remain unknown.

## Discussion

Our current study has several limitations and can be significantly improved in future studies. First, we used drug-SE pairs from SIDER as prior knowledge for the KD approach. The overall performance depends on the accuracy and comprehensiveness of the SIDER database. Errors and uncertainties in the knowledge base can propagate into the relationship extraction process and adversely affect the precision. For example, the drug-disease treatment pair 'ondansetron-pain' was incorrectly specified as a drug-SE pair in SIDER. Because of this error, our algorithm classified the following sentence as SE-related: "**Ondansetron**, lidocaine, tramadol, and fentanyl were effective in preventing and decreasing the level of rocuronium injection **pain**" (PMID 12032018). Three additional pairs (lidocaine-pain, tramadol-pain, and fentanyl-pain) were incorrectly extracted as drug-SE pairs. Since SIDER was constructed from FDA drug labels using text-mining approaches, errors may be inevitable for completely automatic method. Currently, we are manually extracting drug-associated side effects from FDA drug labels. Second, our algorithm cannot extract correct pairs from sentences with multiple drugs and multiple side effect names (n × m), even though the sentences are side effect-related. For example, sentence "... **decreases in hemoglobin**, **nausea/vomiting**, and **hyperbilirubinemia **were observed to be influenced by the previous use of **irinotecan **(OR = 3.07, P = 0.003), **mitomycin **(OR = 2.28, P = 0.004), and **cisplatin **(OR = 1.60, P = 0.007), respectively" (PMID: 17577624). Three drugs and three SEs are specified in the sentence, but only three, instead of 9 (3 × 3) are valid drug-SE pairs. This is a difficult problem for not only the KD approach but also for automatic relationship extraction in general. In this case, human curation may be necessary. Even with the above mentioned limitations, we demonstrated that the large number of drug-SE pairs extracted from MEDLINE reflect drug-related genetic, genomic and chemical information and can have potential in computational drug target discovery and drug repositioning. Currently, we are developing integrative systems approaches for drug repositioning by fully exploiting data ranging from lower level genetic connections to immediate layer genomic data to higher level phenotype data in order to build integrative models of genetic, genomic, and phenotypic complexity.

## Conclusions

We have developed a novel KD approach in extracting a total of 49,575 drug-SE pairs from 119,085,682 MELDINE sentences and 180,454 pairs from 21,354,075 MEDLINE abstracts (records). We show that the KD approach performed significantly better that a SVM-based machine approach. We demonstrated that this large-scale drug-SE association database that we have built provides an invaluable data resource for computational drug target discovery and drug repositioning.

## Competing interests

The authors declare that they have no competing interests.

## Authors' contributions

Xu and Wang have jointly conceived the idea, designed and implemented the algorithms and prepared the manuscript.

## Funding

RX is funded by Case Western Reserve University/Cleveland Clinic CTSA Grant (UL1 RR024989), the Eunice Kennedy Shriver National Institute Of Child Health & Human Development of the National Institutes of Health under Award Number DP2HD084068, the Training grant in Computational Genomic Epidemiology of Cancer (CoGE) (R25 CA094186-06), and Grant #IRG-91-022-18 to the Case Comprehensive Cancer Center from the American Cancer Society. QW is partly funded by ThinTek LLC.

## Declarations

Publication charges for this article have been funded by the Training grant in Computational Genomic Epidemiology of Cancer (CoGE) (R25 CA094186-06).

This article has been published as part of *BMC Bioinformatics *Volume 16 Supplement 5, 2015: Selected articles from the 10th International Symposium on Bioinformatics Research and Applications (ISBRA-14): Bioinformatics. The full contents of the supplement are available online at http://www.biomedcentral.com/bmcbioinformatics/supplements/16/S5.
